# METformin for the MINimization of Geographic Atrophy Progression (METforMIN): A Randomized Trial

**DOI:** 10.1016/j.xops.2023.100440

**Published:** 2023-12-04

**Authors:** Liangbo Linus Shen, Jeremy D. Keenan, Noor Chahal, Abu Tahir Taha, Jasmeet Saroya, Chu Jian Ma, Mengyuan Sun, Daphne Yang, Catherine Psaras, Jacquelyn Callander, Christina Flaxel, Amani A. Fawzi, Thomas K. Schlesinger, Robert W. Wong, Loh-Shan Bryan Leung, Alexander M. Eaton, Nathan C. Steinle, David G. Telander, Armin R. Afshar, Melissa D. Neuwelt, Jennifer I. Lim, Glenn Yiu, Jay M. Stewart

**Affiliations:** 1 Department of Ophthalmology, University of California, San Francisco, San Francisco, California; 2 Francis I. Proctor Foundation for Research in Ophthalmology, University of California, San Francisco, San Francisco, California; 3 Institute of Cardiovascular Diseases, Gladstone Institute, San Francisco, California; 4 Department of Otolaryngology-Head and Neck Surgery, University of California, San Francisco, San Francisco, California; 5 Department of Ophthalmology, Casey Eye Institute, Oregon Health & Science University, Portland, Oregon; 6 Department of Ophthalmology, Feinberg School of Medicine, Northwestern University, Chicago, Illinois; 7 North Bay Vitreoretinal Consultants, Santa Rosa, California; 8 Austin Retina Associates, Austin, Texas; 9 Department of Ophthalmology, Stanford University School of Medicine, Palo Alto, California; 10 Retina Health Center, Fort Myers, Florida; 11 California Retina Consultants, Santa Barbara, California; 12 Retinal Consultants Medical Group, Sacramento, California; 13 Department of Ophthalmology and Visual Sciences, University of Illinois at Chicago, Chicago, Illinois; 14 Department of Ophthalmology & Visual Sciences, UC Davis Medical Center, Sacramento, California

**Keywords:** Age-related macular degeneration, Geographic atrophy, Metformin, Randomized controlled trial

## Abstract

**Purpose:**

Metformin use has been associated with a decreased risk of age-related macular degeneration (AMD) progression in observational studies. We aimed to evaluate the efficacy of oral metformin for slowing geographic atrophy (GA) progression.

**Design:**

Parallel-group, multicenter, randomized phase II clinical trial.

**Participants:**

Participants aged ≥ 55 years without diabetes who had GA from atrophic AMD in ≥ 1 eye.

**Methods:**

We enrolled participants across 12 clinical centers and randomized participants in a 1:1 ratio to receive oral metformin (2000 mg daily) or observation for 18 months. Fundus autofluorescence imaging was obtained at baseline and every 6 months.

**Main Outcome Measures:**

The primary efficacy endpoint was the annualized enlargement rate of the square root-transformed GA area. Secondary endpoints included best-corrected visual acuity (BCVA) and low luminance visual acuity (LLVA) at each visit.

**Results:**

Of 66 enrolled participants, 34 (57 eyes) were randomized to the observation group and 32 (53 eyes) were randomized to the treatment group. The median follow-up duration was 13.9 and 12.6 months in the observation and metformin groups, respectively. The mean ± standard error annualized enlargement rate of square root transformed GA area was 0.35 ± 0.04 mm/year in the observation group and 0.42 ± 0.04 mm/year in the treatment group (risk difference = 0.07 mm/year, 95% confidence interval = −0.05 to 0.18 mm/year; *P* = 0.26). The mean ± standard error decline in BCVA was 4.8 ± 1.7 letters/year in the observation group and 3.4 ± 1.1 letters/year in the treatment group (*P* = 0.56). The mean ± standard error decline in LLVA was 7.3 ± 2.5 letters/year in the observation group and 0.8 ± 2.2 letters/year in the treatment group (*P* = 0.06). Fourteen participants in the metformin group experienced nonserious adverse events related to metformin, with gastrointestinal side effects as the most common. No serious adverse events were attributed to metformin.

**Conclusions:**

The results of this trial as conducted do not support oral metformin having effects on reducing the progression of GA. Additional placebo-controlled trials are needed to explore the role of metformin for AMD, especially for earlier stages of the disease.

**Financial Disclosure(s):**

Proprietary or commercial disclosure may be found in the Footnotes and Disclosures at the end of this article.

Geographic atrophy (GA) is the advanced stage of nonneovascular age-related macular degeneration (AMD), affecting > 5 million patients worldwide.^1^ Geographic atrophy is characterized by progressive degeneration of photoreceptors, retinal pigment epithelium, Bruch’s membrane, and choriocapillaris in the setting of extracellular deposits.^2^ The exact mechanisms for GA progression are unclear, but complement dysregulation, oxidative stress, and mitochondrial dysfunction are among possible pathophysiologic mechanisms that contribute to GA progression.^3^^,^^4^ The United States Food and Drug Administration recently approved intravitreal pegcetacoplan (a complement C3 inhibitor) and avacincaptad pegol (a complement C5 inhibitor) for slowing GA progression based on the results of the OAKS and DERBY phase III trials (ClinicalTrials.gov identifier: NCT03525613 and NCT03525600) and the GATHER2 phase III trial (ClinicalTrials.gov identifier: NCT04435366).^5^^,^^6^ Development is ongoing for treatments that offer greater efficacy and a reduced risk of developing neovascular AMD. An oral agent might decrease the necessity for regular intravitreal injections associated with these complement inhibitors.

Metformin is a potent antihyperglycemic oral medication for treating type 2 diabetes mellitus. Metformin might reduce the progression of GA through multiple biological pathways. Metformin has been shown to inhibit the expression of the nuclear factor-κB gene, leading to a reduction in inflammation.^7^, ^8^, ^9^, ^10^ Moreover, metformin inhibits the mitochondrial respiratory complex I, thereby attenuating the generation of toxic reactive oxygen species by mitochondria.^10^, ^11^, ^12^ Additionally, metformin downregulates the mammalian target of the rapamycin signaling pathway and activates autophagy.^10^^,^^13^ Metformin also has an excellent safety profile, with the most common side effect being gastrointestinal distress, which can usually be overcome via stepwise dose increases.^14^ Metformin has a very low risk for hypoglycemia.^15^ Lactic acidosis is a serious but rare side effect of metformin, with an estimated incidence of 6 cases per 100 000 patient-years.^16^

Given metformin’s broad spectrum of action and good safety profile, it has gained increasing attention as a potential therapeutic agent in treating ocular diseases, including glaucoma,^17^ uveitis,^18^ diabetic retinopathy,^19^ AMD,^20^, ^21^, ^22^, ^23^, ^24^, ^25^ and retinitis pigmentosa^26^. Several recent retrospective studies have found a significant association between metformin use and decreased risk of AMD in patients with diabetes,^20^, ^21^, ^22^, ^23^ but such associations were not significant in some other studies^24^^,^^25^^,^^27^. To our knowledge, no randomized trials have been done to investigate the effect of metformin on AMD, either among diabetic or nondiabetic patients with GA. Therefore, we initiated the METformin FOR the MINimization of GA progression (METforMIN) study (ClinicalTrials.gov identifier: NCT02684578; Registration date: 2/18/2016), a parallel-group, multicenter, randomized phase II clinical trial aiming to evaluate the efficacy of oral metformin in slowing the progression of GA secondary to nonexudative AMD.

## Methods

### Study Design

The study was conceived as an unfunded exploratory trial, a similar approach to previously reported patient-funded studies.^28^ We recruited nondiabetic participants from 12 clinical sites in the United States. We randomly assigned participants in a 1:1 ratio to observation or 18 months of 1000 mg oral metformin hydrochloride twice daily. A research coordinator generated the random allocation sequence stratified by clinical site but without other restrictions. The institutional review board at each clinical site approved the clinical trial, and all participants provided written informed consent at enrollment. We conducted the study according to the tenets of the Declaration of Helsinki, and complied with the Health Insurance Portability and Accountability Act. The clinical trial was overseen by an independent Data and Safety Monitoring Board.

Participants assigned to the metformin group took a dose of 500 mg oral metformin once daily for the first week, 500 mg twice daily for the second week, and then 1000 mg twice daily until 18 months. This stepwise increase in dose was instituted to mitigate gastrointestinal side effects.^14^ Participants were not masked. As the study was not funded, the study investigator at each clinical site prescribed oral metformin hydrochloride (formulation chosen by the investigator) to patients randomized to the metformin group, who obtained the medication at their pharmacies. The participants or their insurance carriers assumed the responsibility of paying for the metformin prescription for the entirety of the study duration. Clinical coordinators called the participants 1 week after the enrollment visit to ensure they had obtained metformin. Participants assigned to the observation group received no specific treatment as part of the trial and were managed according to the standard of care. If a participant developed choroidal neovascularization for which their ophthalmologist advised treatment, the participant exited the study.

### Participants and Eligibility Criteria

A full list of inclusion and exclusion criteria is provided in Table S1 (available at https://www.ophthalmologyscience.org). In brief, eligible participants were nondiabetic, ≥ 55 years of age, and had GA secondary to nonexudative AMD in 1 or both eyes. Geographic atrophy was defined as ≥ 1 well-defined patches of the retinal pigment epithelium absence ≥ 175 μm in diameter on fundus autofluorescence (FAF) imaging, without neovascular AMD. Additional eligibility criteria included a clear ocular media and adequate pupillary dilation, total GA area between 1.25 and 17.5 mm^2^, the ability to photograph the entirety of the GA lesion(s), and best-corrected Snellen visual acuity (BCVA) of 20/20 to 20/400. The study investigator at each clinical site determined the eligibility of patients based on patients’ reports and review of relevant medical records. The investigator determined the eligibility of eyes based on clinical exam, FAF imaging, and additional fundus imaging of the individual investigator’s choice. If both eyes of a participant met the inclusion criteria, we enrolled both eyes in the study.

Exclusion criteria for a participant included current metformin use for another purpose, type 1 or 2 diabetes, compromised renal function, moderate to severe heart failure, Child’s class C cirrhosis, pregnancy, inability to consent, or excessive alcohol use. Exclusion criteria for eyes included evidence of retinal atrophy secondary to causes other than AMD, choroidal neovascularization, and any other ocular disorders that could confound study outcome measures (e.g., diabetic retinopathy, branch or central retinal vein or artery occlusion, macular hole, or pathologic myopia).

### Outcome Measures and Assessments

We evaluated all participants at baseline and every 6 months with a complete ophthalmic examination. Participants in the metformin group were asked to report their compliance level (0%–24%, 25%–49%, 50%–74%, 75%–99%, and 100%) during interviews at each follow-up visit. Each exam included BCVA using the ETDRS chart and manifest refraction, low luminance visual acuity (LLVA) using a 2.0 log unit neutral density filter, color fundus photography, FAF imaging, and OCT. Fundus autofluorescence and OCT images were obtained with the Heidelberg Spectralis device with BluePeak autofluorescence (Heidelberg Engineering) using a prespecified imaging protocol. Adverse events in patients who received metformin were monitored by each clinical site’s investigator through interviews and reviews of medical records at each visit. The investigators also assessed the seriousness, severity, causality, and potential relatedness to the study drug for each adverse event.

The prespecified primary efficacy endpoint was the annualized growth rate of the square root-transformed GA area (mm/year) in the study eye measured from FAF images. Previous studies demonstrated that square root transformation of GA area reduces the primary endpoint’s dependence on baseline lesion size, improving statistical power.^5^^,^^29^, ^30^, ^31^, ^32^, ^33^, ^34^, ^35^, ^36^, ^37^, ^38^ Each FAF image was graded by 2 independent graders masked to treatment allocation using the Heidelberg Eye Explorer software, and the process was detailed in the Supplemental Methods, available at https://www.ophthalmologyscience.org. The graders measured the total area of GA lesions by manually tracing GA borders, and assessed FAF images for GA lesion focality (unifocal vs. multifocal)^39^ and FAF pattern (group 1: “None” or “Focal;” group 2: “Banded,” “Patchy,” or “Diffuse”).^40^^,^^41^ Graders assessed OCT images for GA involvement of the foveal center point. We calculated the mean GA area between 2 graders for each FAF image. Secondary outcomes included the annualized change in BCVA (letters/year), annualized change in LLVA (letters/year), and adverse events in the metformin group.

### Statistical Analysis

We estimated the sample size assuming the mean ± standard deviation of the change in the square root of GA area was 0.55 ± 0.30 mm over 18 months.^42^ Given this mean and standard deviation, 90 participants with GA (45 per group) would provide 80% power to determine an effect size of 33% (i.e., 0.55 vs. 0.37; 2-sided α of 0.05). With additional assumptions of a 10% drop-out rate and only 1 eligible eye per participant, the total sample size goal was 100 participants, with 50 participants per group.

We performed statistical analyses using R 4.0.4 (R Foundation for Statistical Computing). The primary efficacy and safety analyses included all eligible eyes with valid GA area measurements in the baseline visit (intention-to-treat analysis). If a visit did not record the ETDRS letter score, we converted the Snellen visual acuity to the ETDRS letter scores.^43^ We performed the conversion for 10.6% BCVA measurements and 9.5% LLVA measurements. We assessed the intergrader reproducibility of the GA area between each pair of graders via Bland-Altman plots and intraclass correlation coefficients.

We analyzed the primary efficacy endpoint using a repeated measures linear mixed-effects regression model (“lme4” package^44^ in R software) of eye-level data that included all available GA sizes collected over the 18-month treatment period. We modeled the square root of GA area as a function of time from baseline, study group, the interaction term between these 2 variables, and baseline square root of GA area, with random intercepts for eye (to account for repeated measures in the same eye) and participant (to account for the correlation of eyes from the same person) and a random slope for eye across study visits, and incorporating inverse probability of censoring weighting to account for the possibility of selective loss to follow-up. We estimated the probability of censoring after the baseline visit in a multivariable logistic regression model with the following covariates: baseline age, gender, race, bilateral GA status, baseline BCVA in the better eye, and history of medical conditions.^20^ We also created similar mixed-effects models for the secondary outcomes. To investigate the power characteristics of the clinical trial, we used “longpower”^45^ package in R software to calculate the number of eyes needed per group to provide an 80% power (2-sided α of 0.05) to detect a 33% reduction in the square root transformed GA area based on the GA growth rate data in the observation group of this clinical trial.

## Results

### Participant Characteristics

**Figure 1:**
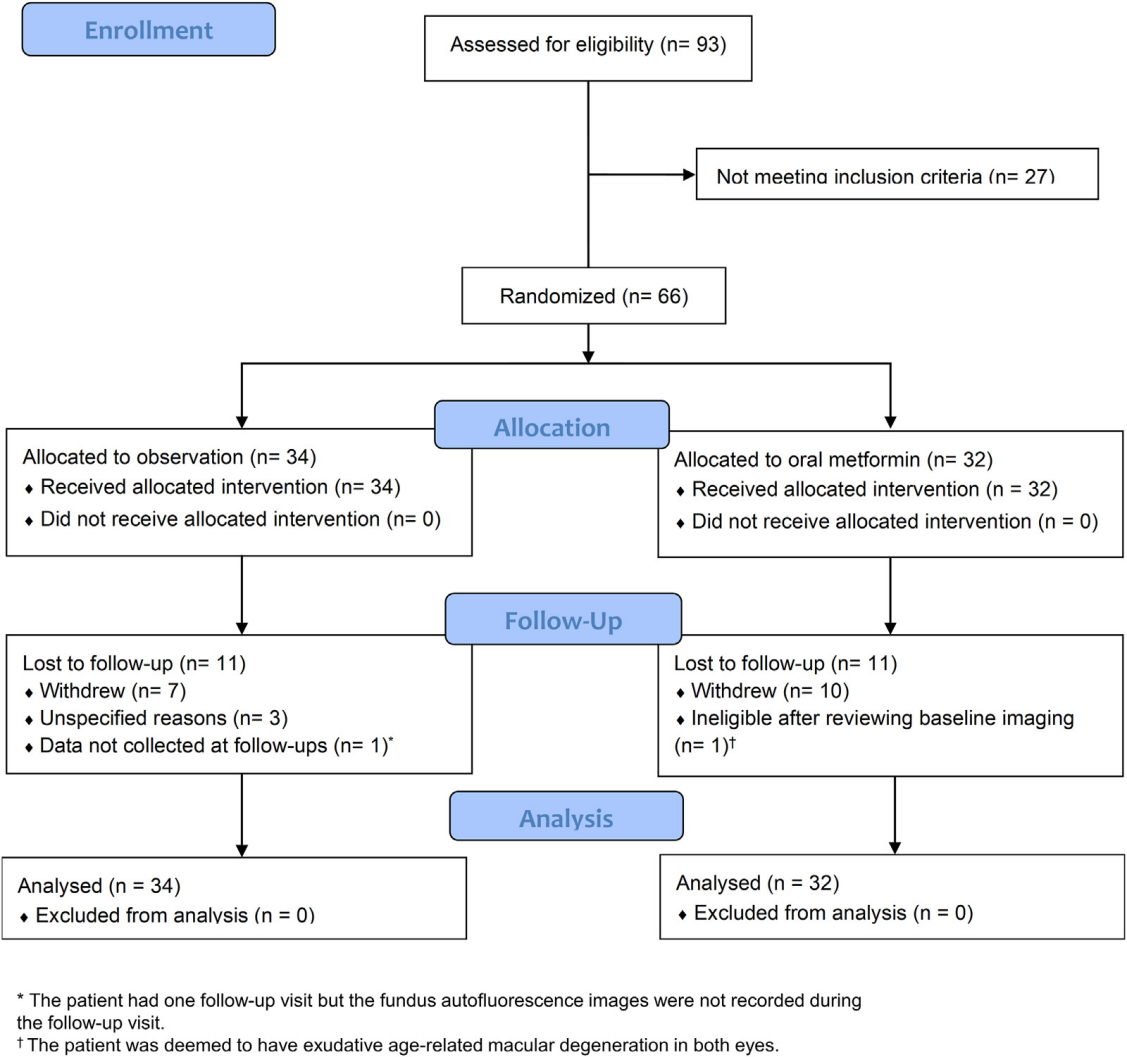
Clinical trial flowchart.

Of 93 participants screened between October 2016 and August 2021, 66 eligible participants were randomized to either observation (34 participants, 57 eyes) or oral metformin treatment (32 participants, 53 eyes) (Fig 1). Our study did not meet its enrollment goal of 50 participants per group due to a lack of study funding. Baseline demographics, medical conditions, and ocular characteristics were generally comparable between the observation and metformin groups (Table 2), although the observation group had a higher mean baseline GA area than the treatment group (mean ± standard deviation: 8.7 ± 6.1 vs. 6.2 ± 4.4 mm^2^). The median follow-up duration was 13.9 months in the observation group and 12.6 months in the metformin group, and the percentage of patients followed at 18 months was 50.0% in the observation group and 40.6% in the metformin group. Among those enrolled, 23 participants (37 eyes) out of 34 participants (67.7%) in the observation group and 21 participants (34 eyes) out of 32 participants (65.6%) in the metformin group had GA area measurements at the baseline visit and ≥ 1 follow-up visit. Eleven participants per group did not contribute follow-up data, including 7 in the metformin group and 10 in the observation group who withdrew. Reasons for withdrawal included the inability to tolerate side effects (6 participants), lack of transportation (2 participants), electing to take metformin from the primary care physicians instead of observation (2 participants), death (2 participants), and other (5 participants). Analysis of the baseline characteristics provided no evidence of differential loss to follow-up between the 2 treatment groups (Table S3, available at https://www.ophthalmologyscience.org). In the metformin group, 88.2%, 94.1%, and 100% of participants reported ≥ 75% compliance with taking metformin at months 6, 12, and 18. No participant in the observation group received treatment for AMD during the 18-month interventional study period. The post hoc power analysis showed that 40 eyes per group were required to provide 80% power (2-sided α of 0.05) to detect a 33% reduction in the square root transformed GA area based on the GA growth rate data in the observation group.

**Table 2:** Baseline Characteristics of Enrolled Participants

	Observation	Metformin
Participants, n	34	32
Age, years, mean (SD)	79.3 (7.3)	78.5 (10.9)
Female sex, n (%)	19 (55.9)	19 (59.4)
White race, n (%)	30 (88.2)	29 (90.6)
Presence of GA in both eyes, n (%)	32 (94.1)	27 (84.4)
Cardiovascular disease, n (%)	22 (64.7)	19 (59.4)
Eyes, n∗	57	53
BCVA, letters, mean (SD)	57.0 (19.4)	58.5 (18.5)
LLVA, letters, mean (SD)†	40.0 (17.9)	42.8 (16.4)
GA area, mm^2^, mean (SD)	8.7 (6.1)	6.2 (4.4)
Multifocal lesion, n (%)	33 (57.9)	34 (64.2)
Foveal center point involvement, n (%)	48 (84.2)	45 (84.9)
FAF pattern, “None” or “Focal,” n (%)	34 (59.6)	38 (71.7)

BCVA = best corrected visual acuity; FAF = fundus autofluorescence; GA = geographic atrophy; LLVA = low luminance visual acuity; SD = standard deviation.

∗ One eye in the metformin group was found to have exudative age-related macular degeneration after reviewing baseline OCT images and was excluded from the table.

† Ten eyes in the observation group and 6 eyes in the metformin group did not have baseline LLVA.

### Efficacy

**Figure 2:**
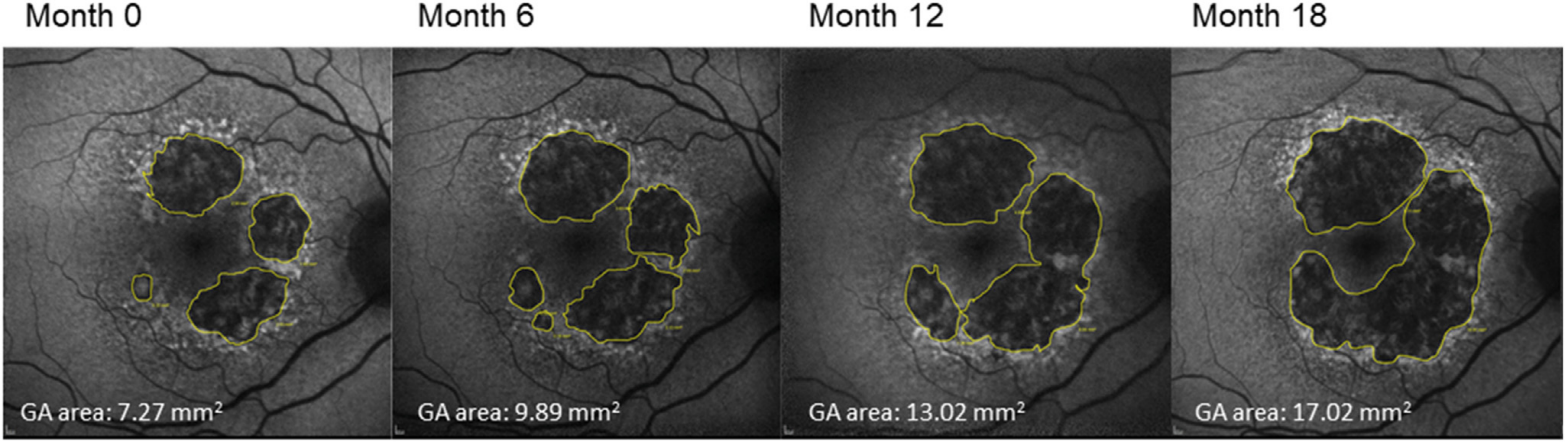
Demonstration of geographic atrophy (GA) grading on the fundus autofluorescence images for 1 representative eye at month 0, 6, 12, and 18. We manually delineated GA borders (yellow lines) and calculated the total area of GA lesions at each visit.

Figure 2 demonstrates the manual delineation of GA lesions in a representative eye based on the FAF images from baseline to 18 months. We found excellent intergrader reproducibility of the square root of GA area between each pair of graders (overall intraclass correlation coefficients = 0.99; Fig S3, available at https://www.ophthalmologyscience.org).

**Figure 4:**
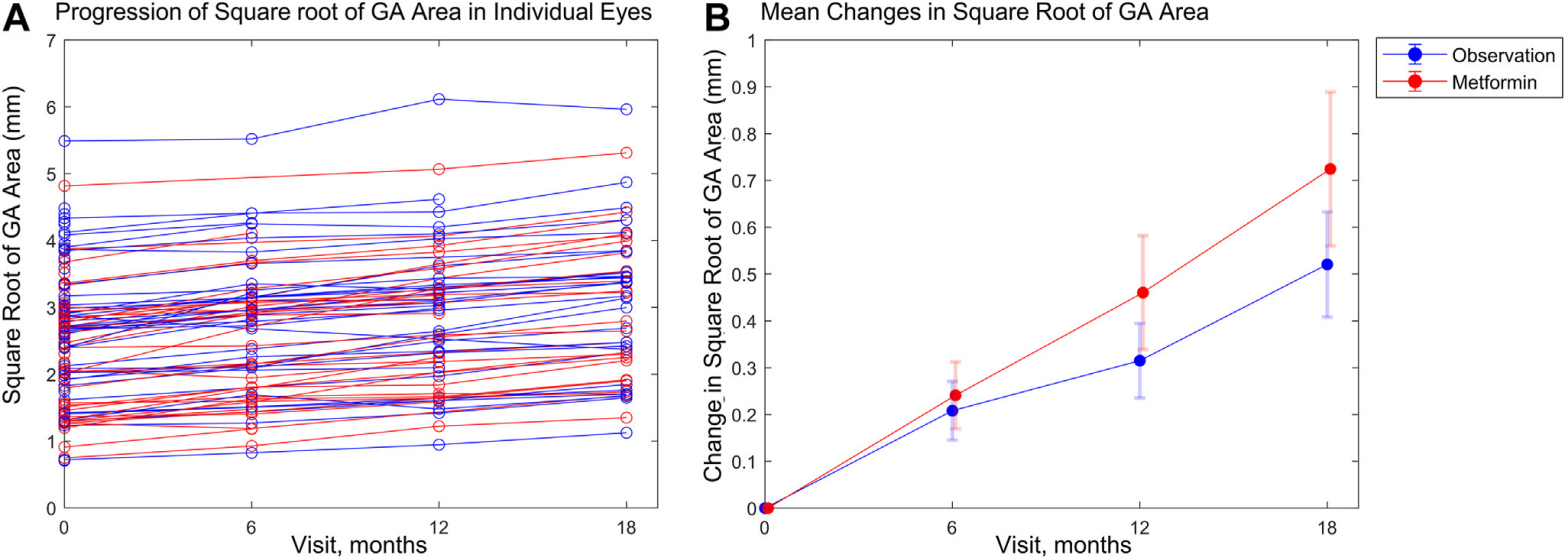
**A,** Progression in the square root of geographic atrophy (GA) area by visit (0, 6, 12, 18 months) in individual eyes. **B,** Mean changes in the square root of GA area (mean ± 95% confidence interval). Number of eyes = 57, 35, 30, and 29 in the observation group and 53, 31, 29, and 24 in the metformin group.

The mean ± standard error growth rate of the square root of GA area from baseline to 18 months (i.e., the primary endpoint) was 0.35 ± 0.04 mm/year in the observation group and 0.42 ± 0.04 mm/year in the metformin group (Fig 4) with a risk difference of 0.07 (95% confidence interval = −0.05 to 0.18) mm/year (*P* = 0.26; prespecified primary analysis).

Best-corrected visual acuity and LLVA declined over the course of the study in both metformin and observation groups. The mean ± standard error annualized change in BCVA ETDRS letter score from baseline to 18 months was −4.8 ± 1.7 letters/year in the observation group and −3.4 ± 1.1 letters/year in the metformin group, with a risk difference of 1.2 (95% confidence interval = −5.1 to 2.7) letters/year (*P* = 0.56). The annualized change in LLVA ETDRS letter score was −7.3 ± 2.5 letters/year in the observation group and −0.8 ± 2.2 letters/year in the metformin group, with a risk difference of 6.5 (95% confidence interval = −0.1 to 13.2) letters/year (*P* = 0.06).

### Safety

A full list of adverse events from participants in the metformin group is listed in Table 4. The most common adverse events were gastrointestinal, including diarrhea (6 participants), unspecified gastrointestinal discomfort (4 participants), nausea (2 participants), vomiting (1 participant), constipation (1 participant), and abdominal swelling (1 participant). In addition, 4 participants reported lethargy and 2 participants reported dizziness in ≥ 1 follow-up visit. Most of the adverse events were self-resolved after 1 visit, and 17 out of 21 participants in the metformin group did not experience any adverse events lasting > 1 visit. Four serious adverse events occurred in 3 participants from the metformin group, including ascending thoracic aortic aneurysm without rupture, acute kidney injury, hepatic fibrosis of unknown origin, and small bowel obstruction. No serious adverse events were considered to be caused by metformin use.

**Table 4:** Adverse Events in Metformin Group (N = 21 Patients)

	Number of Participants With Events in ≥ 1 Follow-Ups	Number of Participants With Events in ≥ 2 Follow-Ups
Any adverse events related to metformin	14	5
Any serious adverse events	3∗	0
Diarrhea	6	1
Unspecified gastrointestinal discomfort	4	0
Lethargy	4	3
Nausea	2	0
Dizziness	2	2
Headache	1	0
Rash	1	0
Vomiting	1	0
Constipation	1	0
Chills	1	0
Poor appetite	1	0
Low B12	1	0
Abdominal swelling	1	0

∗ Three participants in the metformin group had serious adverse events, including ascending thoracic aortic aneurysm without rupture, acute kidney injury, hepatic fibrosis of unknown origin, and small bowel obstruction. None of the serious adverse events were considered to be caused by metformin use.

## Discussion

To our knowledge, the METforMIN study is the first randomized trial aiming to investigate the effect of oral metformin on the progression of GA. The results of the clinical trial do not support the use of oral metformin having effects on reducing GA progression or change in BCVA or LLVA between participants treated with metformin and those not receiving metformin despite relatively high self-reported adherence in the metformin group. The tolerability of oral metformin was moderate in this nondiabetic population, and 6 out of 32 participants in the metformin group withdrew due to tolerability. Most systemic adverse events (e.g., diarrhea) resolved spontaneously or were managed by temporarily reducing the metformin dosage.

The observed progression rate of square root transformed GA area in the present study was at the upper end of the spectrum compared to previously reported rates in the literature (0.27–0.40 mm/year), although it should be noted that the vast majority of participants in the trial had bilateral GA, which has been found to be associated with higher rates of growth.^32^^,^^34^^,^^46^ The GA growth rate in our observation group (0.35 mm/year) was comparable to those in the sham groups of previous large randomized controlled trials recruiting only bilateral GA participants (0.35 mm/year in the Filly trial^5^, 0.37 mm/year in the Chroma and Spectri trials,^30^ and 0.38 mm/year in the GATHER1 trial^47^).

Interestingly, the annualized change in LLVA ETDRS letter score was lower in the metformin group than in the observation group (−0.8 vs. −7.3 letters/year) with a *P* value of 0.06, approaching statistical significance. This result may suggest a potential protective of metformin on photoreceptors, which have been demonstrated in cell studies and animal models.^26^^,^^48^ However, participants in the observation group did not receive any placebos due to the study’s nature as an exploratory trial. Thus, we cannot disregard the potential influence of the placebo effect on the observed reduction in the decline of LLVA.

The null results of the present trial do not support that oral metformin has benefits in slowing GA progression. However, the clinical trial has several limitations. First, our trial did not meet the enrollment goal of 50 participants in each group, limiting our statistical power. The study was conceived as an unfunded exploratory trial, but without financial support for study coordinators, it became challenging for enrollment sites to continue screening and enrolling potential participants. Second, we cannot rule out the possibility that it may take > 18 months of oral metformin use to result in a significant effect on GA progression. Third, GA area was higher in the observation group than in the metformin group at baseline, but our analysis included baseline GA size as a covariable, and the use of square root transformed GA area as an endpoint reduced the dependence of GA growth rate on baseline lesion size. Fourth, although we found no evidence suggesting differential loss to follow-up between the 2 groups, and our analysis addressed the possibility of selective loss to follow-up through inverse probability of censoring weights, it is still possible that participants lost to follow-up could have been different from those remaining in follow-up. For example, participants with more aggressive GA may have been more likely to drop out from the observation group in pursuit of experimental treatment. Fifth, the dose of 1000 mg twice per day was chosen because it is the usual maintenance dosage for type 2 diabetes and has been shown to be better tolerated than a higher dosage.^49^ It is possible that higher or lower doses would be more effective for slowing GA progression. Sixth, this study enrolled participants who already had ≥ 1.25 mm^2^ of GA. We speculate that it may be difficult for any medication to prevent progression at later stages of nonneovascular AMD, when the disease process may be too far along. It is possible that metformin would be more effective if instituted at an earlier stage of disease.^20^, ^21^, ^22^, ^23^ Additionally, we did not assess the adverse events in the observation group.

As one of the first prospective trials examining the efficacy of metformin in ocular diseases,^12^ our study provides some guidance to future trials repurposing metformin for eye disease. Metformin had an acceptable safety profile in this nondiabetic population. Most systemic adverse events (e.g., diarrhea) resolved spontaneously or were managed by temporarily reducing the metformin dosage. Participants were able to obtain metformin successfully. Future trials of metformin for eye diseases among nondiabetics are thus feasible to better understand the role of metformin in eye diseases.

In conclusion, this trial found an acceptable safety profile of metformin when used by a nondiabetic population but did not detect a difference in GA progression between the metformin and observation groups among participants with established GA lesions measuring ≥ 1.25 mm^2^ in area. It is possible that the AMD treated in this trial was too advanced to respond to therapy, and that treatment would need to be started earlier in the disease process, including AMD patients who either do not have GA or those with GA < 1.25 mm^2^. Additional placebo-controlled, randomized trials would be worthwhile to explore the role of metformin for AMD at earlier stages.
