# Supplementary material for: METformin for the MINimization of Geographic Atrophy Progression (METforMIN): A Randomized Trial
Source: Ophthalmol Sci. 2023 Dec 4;4(3):100440. doi: 10.1016/j.xops.2023.100440 (PMC10810745; doi:10.1016/j.xops.2023.100440)
Supplement: Fig S3 [file mmc1.pdf]

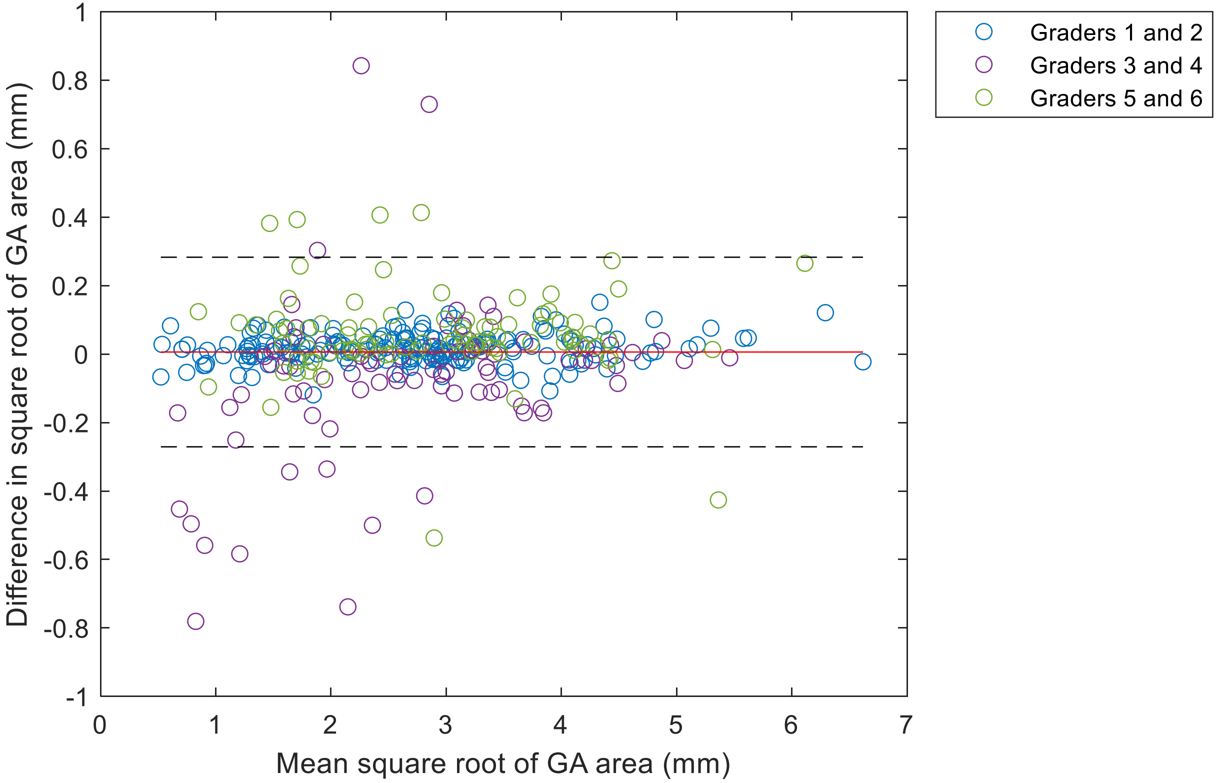

Figure S3. Intergrader reproducibility of the square root area of geographic atrophy (GA) between each pair of graders. Overall, the square root of GA area had a mean difference of 0.006 mm (the solid line) and the 95% limits of agreement of -0.27 to 0.28 mm (dashed lines), with an intraclass correlation coefficient of 0.99 (N = 353 visits).
