# Supplementary material for: METformin for the MINimization of Geographic Atrophy Progression (METforMIN): A Randomized Trial
Source: Ophthalmol Sci. 2023 Dec 4;4(3):100440. doi: 10.1016/j.xops.2023.100440 (PMC10810745; doi:10.1016/j.xops.2023.100440)
Supplement: Supplemental Methods [file mmc2.pdf]

Supplemental Methods. Image Grading Process

A total of 353 fundus autofluorescence (FAF) images were graded by six independent graders for the total area of geographic atrophy (GA) lesions, GA lesion focality (unifocal vs. multifocal) and FAF pattern (group 1: “None” or “Focal”; group 2: “Banded”, “Patchy”, or “Diffuse”). Each image was graded by two independent graders masked to treatment allocation and images obtained at other visits from the same eye. The first 185 collected FAF images were graded by two professional graders (KP and LL) at the University of California, Davis (UCD) reading center. Due to the lack of funding for the study, the remaining 168 images were graded by four trained graders, including three medical students (JS, AT, and NC) and one ophthalmology resident physician (LS), at the University of California, San Francisco (UCSF), using the same grading protocol. To investigate the intergrader reproducibility between UCSF and UCD, we randomly selected 24 images (six images per GA area quartile) that were graded by KP and LL from UCD, and asked each UCSF grader to grade the 24 images. The intraclass correlation coefficient of GA area between each UCSF grader and either UCD grader ranged from 0.94 to 0.98. Then we randomly divided the FAF images not graded by the UCD reading center into image set A and B. JS and AT independently graded each image in image set A, and LS and NC independently graded each image in image set B. The graders also assessed optical coherence tomography images for GA involvement of the foveal center point. Images graded by the three medical students were reviewed by two expert graders (ophthalmology resident physicians: LS and FM) to ensure accuracy: images graded by JS were reviewed by LS and images graded by AT and NC were reviewed by FM. The expert graders corrected GA tracing errors estimated to affect at least 10% of the total GA area measurement. The two expert graders also resolved disagreements between graders in GA presence, lesion focality, FAF patterns, and foveal center point involvement through open arbitration. The total GA area for each FAF image was calculated as the mean GA area between two graders.
