# Supplementary material for: METformin for the MINimization of Geographic Atrophy Progression (METforMIN): A Randomized Trial
Source: Ophthalmol Sci. 2023 Dec 4;4(3):100440. doi: 10.1016/j.xops.2023.100440 (PMC10810745; doi:10.1016/j.xops.2023.100440)
Supplement: Table S1 [file mmc3.pdf]

Table S1. Inclusion and Exclusion Criteria

Inclusion Criteria

- Subject must be >/= 55 years of age
- Subject must have evidence of dry age-related macular degeneration, defined by the characteristic presence of drusen and/or pigmentary changes with or without geographic atrophy. Geographic atrophy (GA) is defined as one or more well-defined and often circular patches of partial or complete depigmentation of the retinal pigment epithelium (RPE) at least 175 µm in diameter and no neovascular AMD, typically with exposure of underlying choroidal blood vessels. Even if much of the RPE appears to be preserved and large choroidal vessels are not visible, a round patch of RPE partial depigmentation may be classified as early GA. The GA in the study eye must be able to be photographed in its entirety, and it must not be contiguous with any areas of peripapillary atrophy, which can complicate area measurements.
- Subject must have clear ocular media and adequate pupillary dilation
- Subject must be able to swallow capsules
- Study eye must have best corrected visual acuity (BCVA) of 20/20-20/400
- Subject must be willing and able to pay for monthly prescription of metformin hydrochloride for 18 months in the event that their insurance carrier will not cover the cost of the drug

Exclusion Criteria

- Subjects with a baseline size of GA < 2.5 or > 17.5mm2.
- Subjects who are already taking metformin for another purpose
- Subjects with type 1 or 2 diabetes
- Subjects with compromised kidney function:
- Serum creatinine ≥1.5 mg/dL for males and ≥1.4 mg/dL for females
- Estimated glomerular filtration rate (eGFR) levels below 45
- Subjects with moderate to severe heart failure (Class III or IV, New York Heart Association Functional Classifications)
- Subjects with Child’s class C cirrhosis
- Subjects who are: pregnant women, adults who cannot consent for themselves, and those who use alcohol in excess
- Excess alcohol use is defined by binge drinking (pattern of drinking that brings blood alcohol concentration levels to 0.08 g/dL) on 5 or more days in the past month
- Evidence of retinal atrophy due to causes other than atrophic age-related macular degeneration.
- Subjects who have had anti-VEGF injections or active choroidal neovascularization in the study eye during the last 12 months
- Current evidence or history of ocular disorders in the study eye that in the opinion of the investigator confounds study outcome measures, including (but not limited to):
    - Branch or central retinal vein or artery occlusion
    - Macular hole
    - Pathologic myopia
    - Uveitis
    - Pseudovitelliform maculopathy
    - Intraocular surgery within the last 90 days prior to study eye enrollment
