# Supplementary material for: METformin for the MINimization of Geographic Atrophy Progression (METforMIN): A Randomized Trial
Source: Ophthalmol Sci. 2023 Dec 4;4(3):100440. doi: 10.1016/j.xops.2023.100440 (PMC10810745; doi:10.1016/j.xops.2023.100440)
Supplement: Table S2 [file mmc4.pdf]

Table S3. Comparisons of Baseline Characteristics by the Loss to follow-up Status

	Not Lost to Follow-up		Lost to Follow-up [*]		
	**Observation**	**Metformin**	**Observation**	**Metformin**	*P*[†]
Participants, n	23	21	11	11	
Age, yrs, mean (SD)	78.7 (6.4)	74.9 (10.7)	80.6 (9.2)	85.5 (7.7)	0.06
Female sex, n (%)	16 (69.6)	13 (61.9)	3 (27.3)	6 (54.5)	0.18
White race, n (%)	21 (91.3)	18 (85.7)	9 (81.8)	11 (100.0)	1.00
Presence of GA in both eyes, n (%)	22 (95.7)	17 (81.0)	10 (90.9)	10.0 (90.9)	0.38
Cardiovascular disease, n (%)	12 (52.2)	12 (57.1)	10 (90.9)	7 (63.6)	0.15
Eyes, n	38[§]	35[§]	19	18	
BCVA, letters, mean (SD)	58.8 (17.4)	61.4 (16.0)	53.4 (22.8)	52.9 (22.1)	0.59
LLVA, letters, mean (SD)[‡]	37.2 (15.0)	44.9 (16.7)	44.7 (21.7)	38.8 (15.6)	0.09
GA area, mm^2, mean (SD)	8.4 (6.3)	6.0 (5.0)	9.4 (5.7)	6.6 (2.9)	0.96
Multifocal lesion, n (%)	25 (65.8)	23 (65.7)	8 (42.1)	11.0 (61.1)	0.37
Foveal center point involvement, n (%)	33 (86.8)	28 (80.0)	15 (78.9)	17 (94.4)	0.54
FAF Pattern, “None” or “Focal”, n (%)	23 (60.5)	25 (71.4)	11 (57.9)	13 (72.2)	0.88

BCVA = best corrected visual acuity; GA = geographic atrophy; LLVA = low luminance visual acuity; SD = standard deviation

[*] Loss to follow-up was defined as patients who had fundus autofluorescence imaging at baseline visits but did not have fundus autofluorescence imaging at any follow-up visits after randomization. Reasons for loss to follow-up are listed in Figure 1.

[†] *P*-value for the interaction term modeling study group by lost to follow-up status.

[‡] 16 eyes did not have baseline low luminance visual acuity.

[§] 1 eye in each group did not have fundus autofluorescence images at any follow-up visit.
